# Challenges in coffee fermentation technologies: bibliometric analysis and critical review

**DOI:** 10.1007/s13197-024-06054-5

**Published:** 2024-09-02

**Authors:** Valeria Hurtado Cortés, Andrés Felipe Bahamón Monje, Jaime Daniel Bustos Vanegas, Nelson Gutiérrez Guzmán

**Affiliations:** Present Address: Facultad de Ingeniería, Grupo de Investigación Agroindustria USCO, Universidad Surcolombiana, Centro Surcolombiano de Investigación en Café – CESURCAFÉ, Avenida Pastrana Borrero Carrera 1a, Neiva, 410001 Huila Colombia

**Keywords:** Postharvest, Sustainability, Prototype, Process control, Processing, Innovation

## Abstract

Advancements in coffee processing technologies have led to improved efficiency in field operations, but challenges still exist in their practical implementation. Various alternatives and solutions have been proposed to enhance processing efficiency and address issues related to safety, standardization, and quality improvement in coffee production. A literature review using SciMAT and ScientoPy software highlighted advancements in fermentation tanks and the emergence of novel fermentation methodologies. However, these innovations lack sufficient scientific evidence. Researchers are now focusing on systematic approaches, such as controlled fermentations and evaluating the influence of microorganisms and process conditions on sensory attributes and coffee composition. Brazil is the leader in coffee bean fermentation research, but the number of published papers in the field has recently decreased. Despite this, efforts continue to improve process control and optimize product quality. The study emphasizes the need for further innovation in coffee fermentation technologies to increase efficiency, sustainability, and profitability while minimizing environmental impact. Implementing these advancements promises a more sustainable and quality-driven future for the coffee industry.

## Introduction

Coffee is a crucial agricultural product and a popular beverage globally. Its growing popularity has led to the need for improved processes to enhance cup quality. The coffee fruit, also known as almond or green coffee, is composed of five layers that protect the endosperm. These layers, known as pulp, mucilage, parchment, and epidermis, form the endosperm and are subjected to roasting to form the flavor and aroma of the coffee drink.

The processing of coffee fruit involves stages to preserve the quality of the almond, which is then subjected to roasting. The types of processing include dry, semi-dry, and wet (de Melo Pereira et al. 2015). Dry processing involves pulping, fermentation, washing, drying, threshing, and roasting. Semi-dry processing involves mechanical removal of the exocarp and part of the mucilage, while wet processing involves drying and threshing.

Green coffee, obtained after processing, varies according to agro-climatological characteristics, species, variety, processing type, and post-harvest operations. Control of these operations is essential to preserve grain quality and maintain the consistency of its sensory profile.

Coffee fermentation is a metabolic process that converts sugars into energy and compounds through the action of enzymes in mucilage (Silva et al. 2013). This process involves the pulped coffee mass being kept in closed containers for 12 to 72 h to remove the mesocarp (mucilage) attached to the parchment. The mucilage, composed of simple sugars and a pectic substrate, is degraded into alcohols and organic acids by microorganisms, such as yeasts and bacteria (Correa et al. 2014; Pereira et al. 2016b).

Coffee fermentation is a metabolic process that converts sugars into energy and compounds through the action of enzymes in mucilage. This process involves the pulped coffee mass being kept in closed containers for 12 to 72 h to remove the mesocarp (mucilage) attached to the parchment. The mucilage, composed of simple sugars and a pectic substrate, is degraded into alcohols and organic acids by microorganisms, such as yeasts and bacteria (Bressani et al. 2021b).

Metabolites diffuse through parchment and endosperm, potentially causing exosmosis during fermentation until chemical potential equilibrium is achieved. The decrease in pH causes mucilage degradation, which can be removed by washing with water. Controlled fermentation can produce beverages with special aromas and flavors, such as sweet, citrus, and fruity.

Mucilage removal can be performed using mechanical or enzymatic methods, such as mechanical abrasion in ELMU-type mucilage removal machines and enzymes like Ultrazym 100, Irgazim 100, Benefax, and Cofepec. Recent research has revealed that the microbial degradation process during fermentation leads to physical and chemical changes in almonds, impacting the sensory characteristics of the resulting drink (da Mota et al. 2020; Elhalis et al. 2021).

Factors affecting reaction rates during coffee fermentation include temperature, water availability, fermentation time, and fruit maturity index. Devices designed for the operation must allow control and measurement of these factors to standardize the process.

This article shows the advances achieved over time with the technologies and methods used in the fermentation of coffee, taking into account the improvement in the processes of safety, standardization and quality of coffee. A bibliographic analysis of research focused on technologies and representative authors with related publications, among other aspects, was carried out. All with the purpose of observing the progress in the fermentation process with the objective of continuing researching and looking for solutions to obtain efficient and quality processing.

## Search methodology

A bibliometric analysis was conducted on coffee bean fermentation publications to identify trends, emerging research directions, and relationships with fermenters and process variables, using Scopus database and ScientoPy and SciMAT software.

### Databases, keywords and search criteria

This study analyzed data from the Scopus database on coffee, bean, and fermentation from 2001 to 2022, focusing on top countries, document types, and institutions, and downloaded in .CSV format for ScientoPy and SciMAT software.

### Analysis using ScientoPy software

The downloaded data from Scopus were then loaded and processed with ScientoPy and the pre-processing results are presented in Table 1. The journals and keywords categories were analyzed with the software to know the most relevant issues, total documents published by journal and keywords and evolution of them in the period from 2001 to 2022.

**Table 1:** Results of data pre-processing in ScientoPy and parameters for the analysis in SciMAT Software

Information	Number
Loaded papers	223
Omitted papers	16
Total papers	223
Duplicated papers found	0
Original papers count	223
Actual papers count	223
Removed papers Scopus	0
**Module for managing the knowledge base**	
Building of knowledge base	SCOPUS
Imported files	.RIS
Documents Pre-processing	Manual removal of unrelated documents Manual search for duplicate documents Manual grouping of similar or duplicate words
Period definition	2001–2017, 2018–2020, 2021–2022
**Module for carrying out scientific mappping analysis**	
Selection periods	2001–2017, 2018–2020, 2021–2022
Selection of unit of analysis	Words: Author words, source’s words
Data reduction	Minimum frequency for all periods: 2
Network type	Co-ocurrence
Network reduction	Minimum value: 2
Normalization	Association strength
Clusters	Maximum network size: 7/ minimum network size:5
Document mapper	Core mapper/secondary mapper
Measurement quality	h-index/sum citation
Longitudinal analysis	Evolution map: Inclusion index/Overlapping map: Jaccard’s index
**Display module**	
Visualization	Longitudinal view/Period view

### Analysis using SciMAT software

The data was preprocessed in modules “Knowledge base” and “Group sets” to remove duplicates and related keywords. The analysis was set to three periods (2001–2017, 2018–2020, and 2021–2022) and parameters were selected in “Analysis” to create evolution maps, strategic diagrams, and clusters.

## Search results

### Trends in publications over time

**Fig. 1:**
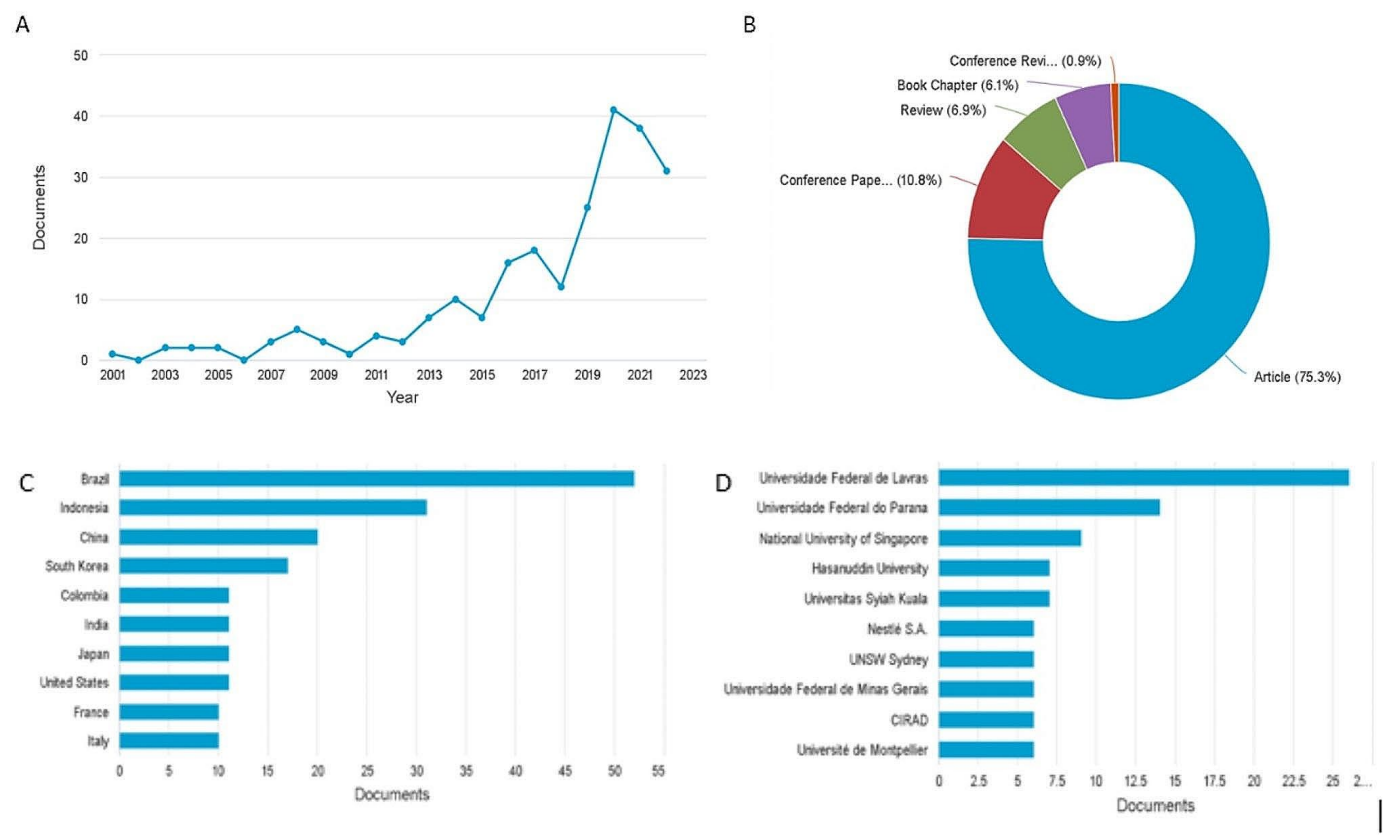
Documents published in the search topic (Graphs extracted from Scopus online database). (**A**) Documents published by year (**B**) Documents published by type (**C**) Documents published by country (**D**) Documents published by institution

The search for 231 documents from 2001 to 2022 revealed a stable trend in published documents. Between 2012 and 2022, there was a decrease in publications (Fig. 1A and B). The top five countries were Brazil, followed by Indonesia, China, South Korea, and Colombia (Fig. 1C). The majority of documents were articles, with a small percentage of reviews. The Universidade Federal de Lavras leads the list with 26 publications on the search topic, followed by Brazilian Universidade Federal do Parana (Fig. 1-D). Indonesian institutions Hasanuddin University and Universitas Sylah Kuala also appear in the top 10. Nestlé S.A. ranks sixth in the top ten.

**Fig. 2:**
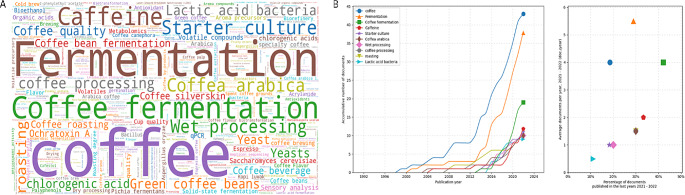
Keywords in research related to the search topic (**A**) Cloud diagram of the top 1000 words (**B**) Evolution graph of the top 10 words

There is no clear trend in the number of publications in the top 10 journals over time. Food Research International and IOP Conference Series: Earth and Environmental Science had the largest publications in 2020 and 2021 respectively. The evolution of the top 10 keywords is showed in Fig. 2. This graph corroborates the main terms used and shows an increase over time of this words. After “Coffee”, which is a general term, “Fermentation” term has the highest number of documents published in the period analyzed, but according to the percentage of documents published in the last year 2021–2022 graph, “coffee fermentation” had the highest value (42.1%) in comparison to the other terms, which indicates that in the last year, this topic has had a higher relative growth.

Although in general the interest in the fermentation process of coffee remains constantly growing, the topics addressed are diverse, which can be evidenced in the variety of key terms that the search throws up. Figure 2-A shows the cloud of words specifically related to the fermentation process, such as types of fermentation, process control, variables involved, and fermenters. In relation to the latter, only two specific terms about it appear in the cloud, “bioreactors” and “bioreactor”; and some terms related with variables, control process or devices such as “controlled fermentation”, “temperature distribution”, ohmic technology”, “ohmic heating”.

Besides, some differences are obtained with the processing of data in SciMAT (Table 2), since this tool allows groups conformations in similar words and documents not related with the topic, however, words like “Fermentation” and “Coffee” remains as main terms, which is expected since these are general terms. Respect to the journals, the results are similar with some exceptions.

**Table 2:** Top ten of the number of published documents in relation to keywords, journals and authors analyzed with ScyMAT

No	Term	Documents	Journal	Documents	Author	Documents
1	Fermentation	90	Food Research International	13	Schwan, R.F.	20
2	Coffee	74	LWT	8	Soccol, C.R.	14
3	Yeast	37	Food Chemistry	6	de Carvalho Neto, D.P.	11
4	Lactic acid	23	Fermentation	6	de Melo Pereira, G.V.	10
5	Metabolism	22	International Journal of Food Microbiology	5	Yu, B.	10
6	Chemistry	21	Food Microbiology	4	Liu, S.Q.	9
7	Nonhuman	20	Frontiers in Microbiology	4	Martinez, S. J.	8
8	Bacteria	20	3rd International Conference on Food Security and Sustainable Agriculture in the Tropics	3	Bressani, A.P.P.	7
9	Yeasts	19	Coffee Science	3	Soccol, V.T.	7
10	Food processing	19	Journal of Korean Society of Food Science Nutrition	3	Batista, N. N.	6

### Topic evolution map and strategic diagram and cluster’s network

**Fig. 3:**
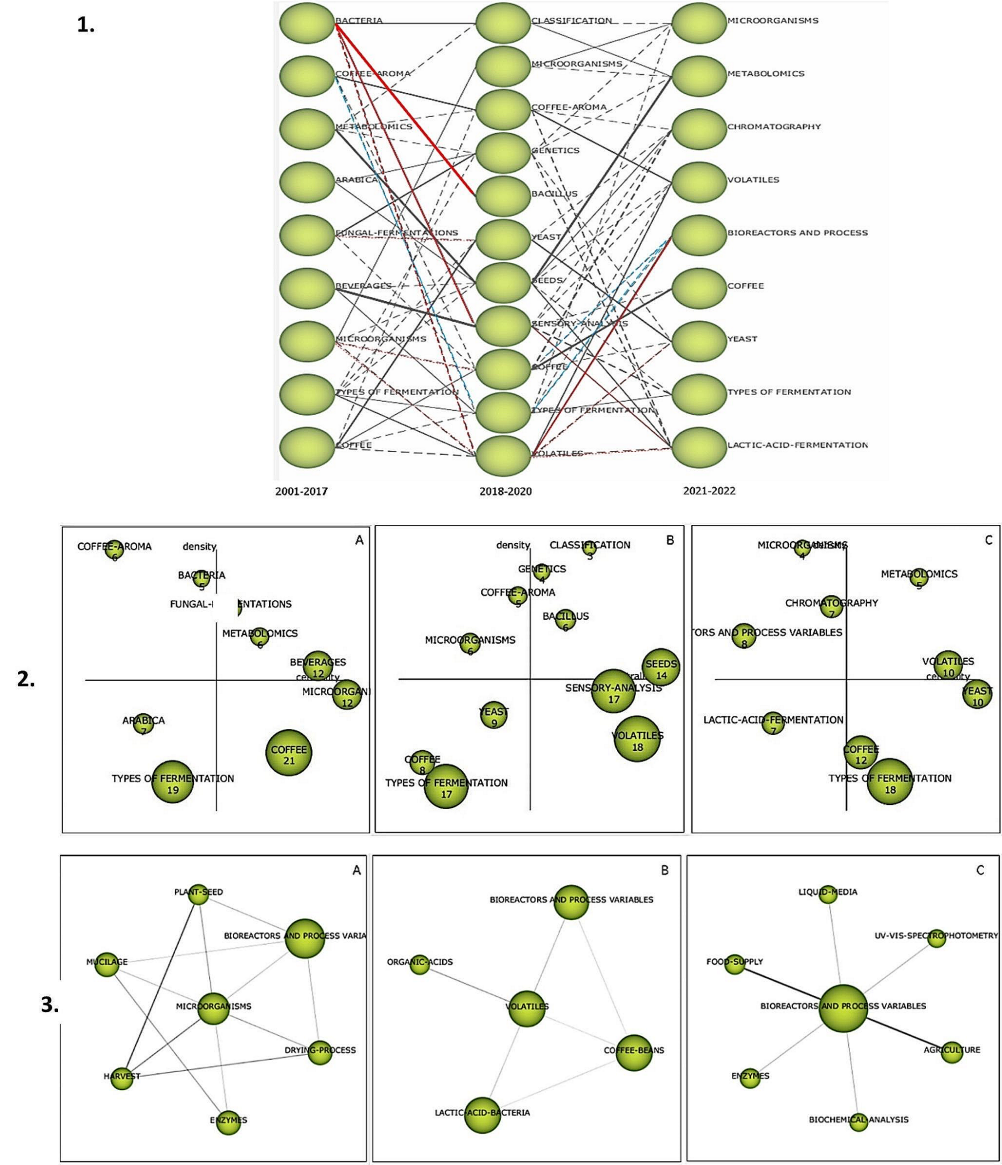
(**1**) Evolution map of the relevant terms regarding the search topic in the documents reported from 2001 to 2022, and relevant relationships on evolution map. The color lines represent the main associations found, and (**2**) Strategic diagram associated with topic of interest for period (**A**) 2001–2017, (**B**) 2018–2020, (**C**) 2021–2022. (**3**) Cluster of terms associated with topic of interest for period (**A**) 2001–2017, (**B**) 2018–2020, (**C**) 2021–2022

Figure 3 shows the graphs generated by SciMAT from the analysis of the data associated with the search string. According to the evolution map (Figs. 3-1), for period 2001–2017 it can be observed nine main terms: “bacteria”, “coffee aroma”, “metabolomics”, “arabica”, “fungal fermentations”, “beverages”, “microorganisms”, “types of fermentation”, and “coffee”. In the period 2018–2020, the number of relevant terms increase to eleven, and only “microorganisms”, “coffee” and “type of fermentation” terms remain. New terms are included such as “classification”, “genetics”, “bacillus”, “yeast”, “seeds”, “sensory analysis” and “volatiles”. With respect to period 2020–2022, “bioreactors and process variables” term appears for the first time and there are new terms, for instance, analytical techniques as “spectrometry” and “chromatography”.

With respect to the relevant associations shown on the evolution map (Figs. 3-1), it is possible to observe terms associated with the study of microorganisms (relationships highlighted in red), such as bacteria, especially of the genus Bacillus, fungal fermentation, mainly related to yeasts, and lactic acid fermentation. These terms, in turn, present associations with parameters such as sensory analysis and volatiles. The latter is related to “bioreactor and process variables” in the last period. The term “bioreactor and process variables” is backward associated with “coffee”, a general term, and “types of fermentation”, which, in turn, is associated with “coffee aroma” (relationships highlighted in blue). These associations are anticipated due to the disparate processing methodologies, where the conditions of the process, the utilization of starter cultures, the type of microorganisms employed, and the generation of organic acids that contribute to alterations in the volatile and aroma profiles of roasted coffee are implicated (da Mota et al. 2020). Additionally the control of process variable offered by bioreactors has recently shown to contribute to the production of coffee with higher sensory quality and reproducibility (de Carvalho Neto et al. 2018).

The strategic diagrams and cluster networks for each period are presented in Fig. 3 (2) and (3). The volume of the spheres is proportional to the number of published documents associated with each theme. The upper-right quadrant is motor-themes, with terms in the upper-left quadrant being highly development and isolated themes. The lower-left quadrant is emerging or declining themes, while the lower-right quadrant is transversal and general. In the first period, “beverages”, “metabolomics”, and “fungal fermentation” are motor themes, while “types of fermentation” is an emergent theme. In the second period, “types of fermentation” remains an emergent theme, with “yeast” added to this category. In the third period, “coffee” is less frequent, with more specific topics such as “bacillus”, “genetics”, and “classification” as motor themes (Elhalis et al. 2021). For the period 2020–2022, “volatiles” and “metabolomics” are motor themes, related to research on volatiles and metobolites generated in process fermentation (Elhalis et al. 2021; Prakash et al. 2022). “Types of fermentation” and “coffee” are tranversal themes, while “lactic acid fermentation” is an emergent theme. “Bioreactors and process variables” is an isolated theme for this period.

Figure 3 (3) displays clusters of terms related to bioreactors and process variables control. The volume of spheres is proportional to the number of documents corresponding to each keyword, and the thickness of the link between two spheres is proportional to the equivalence index between these words. For the period 2001–2017, “bioreactor and process variables” is slightly related to “microorganisms”, “drying process”, “plant seed”, and “mucilage”. For the second period, “volatile” term shows a slight relationship with “bioreactor and process variables”, which in turn is associated with “coffee beans”. The main associations with “bioreactor and process variables” may be with “microorganisms” and “volatile”.

As regards the period 2021–2022 (Figs. 3 - (3) -C), strong bonds are observed between “Bioreactor and process variables”, “Food supply” and “Agriculture”, since the fermentation process is usually on-farm process, although efforts have been made to the process control (Martinez et al. 2017; de Carvalho Neto et al. 2018). Slight associations are seen with more specific terms such as “Liquid media”, “Enzymes”, “UV-VIS-Spectrophotometry” and “Biochemical analysis”. These terms are directly related to the fermentation process and its monitoring, whereby those relationships are expected.

## Coffee fermentation methodologies

Scientific publications related to coffee fermentation devices were searched in the Web of Science, Scopus, and Science Direct databases. Published patents, as well as devices developed by different companies in the sector, were also included in the review. The evolution and characteristics of the devices and their impact on the quality and sensory profile of the coffee were tabulated and summarized in tables.

Table 3 presents various methods for controlling coffee fermentation, focusing on temperature, processing time, and microorganism addition. These parameters affect the grain’s physical-chemical composition and sensory profile. Fermentation times range from 12 h to several days, with low temperatures slowing microbial kinetics and requiring several days for pH to reach 3.8. Mass transfer between mucilage and grain layers occurs mainly through diffusion, leading to more complex profiles in long-time fermentation.

**Table 3:** Methodologies for coffee fermentation

Country / Specie	Variety/ process	Process remarks	Sensory	Physicochemical evaluation	Ref.
Brasil / Arábica	Catuaí Amarelo / wet	- Time: 24 h. - Microoganism: Pichia fermentans YC5.2 and Pediococcus acidilactici LPBC161.	- Overripe fruits can transfer unwanted aromas to the coffee beans. - The production of lactic acid during fermentation contributed to the improvement of the acidity and body of the drink.	- Aromatic compounds ↑: ethyl acetate, isoamyl acetate and ethyl isobutyrate. - Formation of primary metabolites ↑: ethanol and lactic acid. - Initial yeast cultures ↑ and BAL, with consumption ↑: mucilage and aromatic compounds.	(Pregolini et al. 2021)
Brasil /	Bourbon Amarelo and Canário Amarelo / dry	- Four starter cultures: Meyerozyma caribbica (CCMA 0198), Saccharomyces cerevisiae (CCMA 0543), Candida parapsilosis (CCMA0544) and Torulaspora delbrueckii (CCMA 0684).	- Citric, malic, succinic, lactic, oxalic, isobutyric and propionic acids and 105 volatile compounds were detected, improving the fragrance and aroma of coffee. - Improved perception of the sensory attribute (fruity, nutty, cocoa) varied with the coffee variety, process and inoculum.	- The following organic acids were detected: citric, malic, succinic, lactic, oxalic, isobutyric and isovaleric. - ↑ concentrations in green coffee of butanoic, decanoic, hexanoic and propanoic acids.	(Bressani et al. 2020)
Brasil / Arábica	Catuí	- Temperature: 30 °C. - Spontaneous and inoculated fermentation (Lactobacillus plantarum LPBR01). - Lactic acid fermentation. - Fermentation in a stirred tank bioreactor.	- Better aromatic composition. - The aroma, flavor, acidity, body and balance reached higher scores in the inoculated treatment compared to the spontaneous one. - SCA score: 91.5 and 85.5 for the respective inoculated and spontaneous processes.	- ↑ production of lactic acid. - ↓ pH below 4.0 in the first 10 h. - ↓ fermentation time went from 24 h to 10 h.	(de Carvalho Neto et al. 2018)
Brasil / Arábica	Catuaí Amarelo / Dry	- Time: 87 h - Temperature: 30.5–29.67 °C. - Self-induced anaerobic fermentation. - Natural fermentation and natural pulping.	- Classified as specialty coffees, SCA cup score 80 points.	Maximum amount of citric, acetic and lactic acid. Furan chemical class detected.	(Batista da Mota et al. 2022)
Brasil / Arábica	Catuaí Vermelho / Dry	- Time: 24, 48, 72, 96 y 120 h - Temperature: 18, 28 y 38 °C - Process: Carbonic maceration. - Anaerobic conditions.	- SCA score 85 for 38 °C at 120 h of fermentation.	Presence of trigonelin, formic acid, hydroxymethylfurfural, lipids and γ-butyrolactone.	(Brioschi Junior et al. 2021)
Brasil / Arábica	Catuí / wet	- BL Inocularion: Lactobacillus plantarum LPBR01.	- Lactobacillus plantarum LP R01 showed adequate production of organic acids and aromatic esters.	↓ fermentation time from 24 to 12 h. Aromatic compounds: ethyl acetate, ethyl isobutyrate and acetaldehyde.	(Pereira et al. 2016a)
Indonesia / Arábica	Gayo / Dry	- Time: 30 days - Temperature: 25 30 °C - Ph: 5,03,9	No mentioned	Presence of yeasts, lactic bacteria (LAB) and acetic bacteria (AAB).	(Sulaiman and Hasni 2022)
Brasil / Arábica	Catuaí Amarelo / Dry	- Time 16 h - Microorganism: Saccharomyces cerevisiae CCMA 0543, Candida parapsilosis CCMA 0544 y Torulospora delbrueckii CCMA 0684. - Methods: qPCR, HPLC, GC-MS.	- Sensory analysis in roasted coffee. - ↑ concentrations of citric and malic acid. - Concentrations of caffeine, chlorogenic acids and trigonelline and 217 volatile compounds. - Caramel flavor in the samples with Saccharomyces cerevisiae CCMA 0543 D. - Fruity flavor (apple, cherry, banana) in the samples with Candida parapsilosis CCMA 0544 D.	Content of chlorogenic acids, caffeine, trigonelline. Volatile compounds depending on the inoculation method: 1,4-butanediol and 2,6-dimethylpyridine, 3,4-dimethyl-1-pentanol, 4, (2,6,6-trimethyl-2-cyclohexen-1 -yl, -3-buten-2-one, 4-hydroxy-3-methoxy-benzaldehyde and 1,2,3-propanetriol, among others.	(Bressani et al. 2018)
Brasil / Arábica	Mundo Novo y Catuaí / wet	- Microorganism: Saccharomyces cerevisiae CCMA 0200 and Torulaspora delbrueckii CCMA 0684.	- SCA score: 80, 81 and 82 for the treatments S. Cerevisiae CCMA 0200 and T. Delbrueckii CCMA 0684, respectively. - Catuaí: flavors and aromas of caramel, yellow fruits, brown sugar, herbaceous and floral. - Mundo Novo: flavors and aromas of caramel, herbaceous, brown sugar and honey.	- Eight volatile compounds were detected in green coffee and 75 in roasted coffee. - 2-furanmethanol propanoate and 2-ethyl-3,5-dimethylpyrazine were identified only in the inoculated treatments	(Martins et al. 2019)
Brasil / Arábica	Ouro Amarelo and Mundo Novo / Semi-dry	- Microorganism: three yeast strains (Saccharomyces cerevisiae CCMA 0200, CCMA 0543 and Torulaspora delbrueckii CCMA 0684).	- Two sensory analysis techniques: analysis of the flavor in the cup and the temporal dominance of the sensations. - The Ouro Amarelo variety inoculated with CCMA 0543 ↑: acidity and nuts. - The Mundo Novo variety inoculated with the treatments CCMA 0543 and CCMA 0684, ↓: the sensation of astringency.	No mention.	(Ribeiro et al. 2017)
Brasil / Arábica	Mundo Novo / Dry	- Time: 27 h - Temperature: 16,5–24 °C - Microorganism: yeast Meyerozyma caribbica (CCMA 0198), Saccharomyces cerevisiae (CCMA 0543), Candida parapsilosis (CCMA 0544) and Torulaspora delbrueckii (CCMA 0684).	- SCA score 85.75 and 84.92 points. - Lactic, isobutyric and isovaleric acids were detected at the end of fermentation in different treatments. - Scores ↑ for sweetness, long aftertaste and greater complexity.	- Volatile compounds, such as 2,6-diethylpyrazine, in the treatments inoculated with yeasts, but not in the Controls. -2-acetoxymethylfuran was only detected in the samples inoculated with CCMA 0198, both in the NAT and PN methods.	(Bressani et al. 2021b)
Brasil / Arábica	Mundo Novo / wet	- Time: 48 h - Daytime temperature: 23–30 °C - Night temperature: 11–15 °C - Microorganisms: P. Fermentans YC5.2 and Saccharomyces sp. YC9.15.	- Fruity, buttery and fermented aroma.	- 144 isolated yeasts. - ↑ frequent Pichia fermentans and Pichia kluyveri were the isolates, followed by Candida glabrata, quercitrusa, Saccharomyces sp., Pichia guilliermondii, Pichia caribbica and Hanseniaspora opuntiae. - ↑ concentrations of aromatic esters: ethyl acetate and isoamyl acetate.	(de Melo Pereira et al. 2014)
México / Arábica.	Garnica / Dry and wet	- Time: 24 h - Temperature: environment.	Natural: Aroma of red wine. Flower notes of coffee, lemon, fresh butter, dark chocolate, tea rose, caramel, black currant, coriander seed, toasted bread, nuts, vanilla and maple syrup.	No mention	(Partida-Sedas et al. 2019)
Brasil / Arábica	Acaiá / Semi-dry	- Time: 48 h, 312 h, 720 h - Temperature: 28 °C - Microorganism: Saccharomyces cerevisiae UFLA YCN727, S. Cerevisiae UFLA YCN724, Candida parapsilosis UFLA YCN448 and Pichia guilliermondii UFLA YCN731. - Methods: DGGE, HPLC y HS-SPME/GC, TDS.	- Caramel flavour. - Herbaceous sensation. - Light acidity.	Detected compounds: Organic acids: citric, malic, succinic, lactic and acetic. 47 volatile compounds. 3 ketones, 15 alcohols, 5 aldehydes, 7 acids, 10 esters, 5 furans, 1 phenol and 1 diether.	(Evangelista et al. 2014)
Brasil / Arábica	Bourbon amarelo and Catucaí amarelo Rubi / Dry	- Time: 0, 30 y 70 h - Temperature: 18 °C, 22 °C y 30 °C - Microorganism: Saccharomyces cerevisiae (CCMA 0543) y Torulaspora delbrueckii (CCMA 0684) - Methods: GC-MS y HPLC.	- Bourbon, ↑ sensory scores. - Catucai, at a lower altitude, obtained a better sensory score in natural coffee. - ↓ concentration of phenols and ↑ concentration of pyrans. - Scores between 83 and 85, according to the SCA.	Succinic, citric, lactic and acetic acid Volatile groups mainly detected: Ethanol, Pyrrole, furan, alcohol, aldehyde, amine, ketone ester, lactone and thiophene.	(Evangelista et al. 2015)
Brasil / Arábica	Catucaí amarelo / Semi-dry	- Time: 0, 16, 112, 256 and 352 h. - Temperature: ambient (14.6 and 28.2 °C). - Addition of microorganisms: Saccharomyces (S.) Cerevisiae (CCMA 0543), Candida (C.) Parapsilosis (CCMA 0544) and Torulaspora (T.) Delbrueckii (CCMA 0684). - Direct inoculation and bucket. - Methods: HPLC and GC-MS.	- Scores greater than 80, according to the SCCA. - The compounds detected are related to flavor and aroma, such as fruity, sweet, caramel, nutty, and floral flavors and odors, among others.	- Organic compounds detected: citric, malic, succinic, lactic, acetic, propionic, isobutyric and chlorogenic acids, as well as glycerol and ethanol.	(da Mota et al. 2020)
Brasil / Arábica	Catuaí Vermelho / wet	- Time: 72 h. - Room temperature - Microorganisms: Leuconostoc mesenteroides CCMA1105 and Lactiplantibacillus plantarum CCMA 1065 and Saccharomyces cerevisiae CCMA0543 and Torulaspora delbrueckii CCMA0684. - SIAF, autoinduced anaerobic fermentation in Bioreactor.	- Scores ranged from 79.0 to 83.25. - Aroma notes of walnut and almond, floral and dried fruit, and flavor notes of walnut and almond, honey, milk chocolate, tropical fruit, vinous, red fruit, milky notes, caramelised sugar, floral, dried fruit, caramel, spices, yellow fruits, citrus, dark chocolate, orange, woody and cereal.	- Production of lactic and acetic acids. - Chlorogenic acid and trigonelline 2,3-butanediol produced by lactic acid bacteria, which contributes to the aroma profile.	(Martinez et al. 2017)
Brasil / Arábica	Catucaí / wet	- Time: 12–24 h - Temperature: 30 °C. - Microorganisms: Pichia fermentans YC5.2 - Methods: HPLC and GC-MS.	- Grains inoculated with Pichia fermentans received scores higher than 90 points, according to the SCCA.	- Metabolic compounds: organic acids, ethanol and ethyl acetate. - Lactic acid, citric. - Aromatic composition (including D-Limonene, phenylacetaldehyde and phenylethyl alcohol).	(Cassimiro et al. 2022)
Colombia / Arábica	No mention / wet	- Time: 48 h. - Room temperature - Methods: HPLC y GC-MS.	- Floral, fruity and citrus sensory notes.	- Lactic acid and acetaldehyde were the main final metabolites. - 20 volatile compounds were produced, including alcohols, organic acids, aldehydes, esters, terpenes, phenols, and hydrocarbons.	(Carvalho Neto et al. 2020)
Australia / Arábica	Bourbon / wet	- Time: 36 h. - Room temperature (18 °C). - Methods: HS-SPME/GC-MS.	- Coffee made with fermented beans received a rating of ↑ in flavor, aroma, acidity, body and uniformity. - Fruity aroma (apple, yellow grape), with plum, vanilla. Taste of brown sugar and toasted nuts, with notes of cinnamon, shallot and vegemite.	- ↑ concentrations of ethanol, isoamyl alcohol, 3-methylbutanal, benzaldehyde, acetaldehyde and ethyl acetate. - Presence of: aldehydes, esters, ketones, phenols, organic acids and pyrazines.	(Elhalis et al. 2021)
Panamá / Arábica	Geisha / wet	- Time: 48 and 60 h. - Temperature: ambient (21 °C). - Microorganisms: Saccharomyces cerevisiae - CO2 injection - Methods: HPLC.	- Scores ranged from 89.49 to 92.22. - Flavors of kiwi, watermelon, honey and peaches.	- Higher content of total chlorogenic acids at 48 h.	(Samaniego Rodriguez 2019)
Brasil / Arábica and robusta	Catuaí Vermelho and Robusta Vitoria / wet and dry	- Time: 36 h. - Temperature: 38 °C. - Microorganisms: Saccharomyces cerevisiae. - Methods: FTIR	- SCA score: Arabica coffee 86.4 points. - SCA Score: Robusta coffee 80.9 points.	- Chlorogenic acids. - Presence of sugars and carboxylic acids.	(Fioresi et al. 2021)
Brasil / Arábica	Catuaí Vermelho / Dry	- Time: 72 and 672 h. - Temperature: 18–24.5 °C. - Matrix Assisted Laser Desorption Ionization Mass Spectrometry (MALDI-TOF MS).	- Floral attribute was detected only at the altitude of 1400 m. - Caramel, chocolate and almond attributes were detected at higher altitudes.	- Presence of chlorogenic acid and fatty acids. - AF such as palmitic, stearic and arachidic are potential discriminators of the sensory quality of specialty coffees.	(Martins et al. 2020)
India / Robusta and congensis	Coffea congensis and Coffea canephora / Dry	- Time: 52 and 60 h. - Microorganism: Saccharomyces cerevisiae	No mention	- The changes in the content of alkaloids and chlorogenic acid were insignificant. - Presence of: aldehydes, alcohols, fatty acids and carboxylic acids, pyrazines and furans	(Prakash et al. 2022)
Brasil / No mention	Catuaí Vermelho / Dry	- Time: 72 h. - Temperature: 21,9 °C. - Microorganisms: Saccharomyces cerevisiae CCMA 0543, Candida parapsilosis CCMA 0544, or Torulaspora delbrueckii CCMA 0684). - Methods: PCR	- Sensory scores greater than 86 points. - Fruity, citrus, molasses, freshness and wine notes.	- Organic acids and volatile compounds	(Bressani et al. 2021a)

## Evolution of technologies for coffee fermentation

Coffee fermentation devices are containers that allow the product volume to be maintained under homogeneous conditions during the process. Fermentation can be done dry or submerged, with the latter ensuring that all grains are in contact with an equal volume of oxygen. Most producers follow this method for a more homogeneous fermentation. Initially, pulped coffee was fermented in vat-type tanks made of wood, cement, or brick, which were plastered or enameled with cement or covered with baked clay veneers. The floor was built with a slope of 6 to 8% and a width-to-height ratio of 1:1.5 to facilitate leachate drainage and product removal. The final processing time was determined based on producer experience, such as the hole and touch test, which is subjective and prone to errors.

Producers have noticed that fermentation under certain conditions can result in heterogeneous coffee products with sour and fermented flavors due to the difficulty in controlling process variables. To improve sanitary conditions, Cenicafé and other manufacturers have developed high-density polyethylene vat-type tanks, which are lightweight, easy to handle, and clean. Cenicafé in Colombia has successfully maintained a stabilized process with less washing water consumption using Ecomill^®^ technology in stainless steel and high-density polyethylene. These systems incorporate cylindrical fermentation tanks with inverted cone-shaped bases and 60° horizontal inclination, allowing coffee to flow out by gravity.

**Fig. 4:**
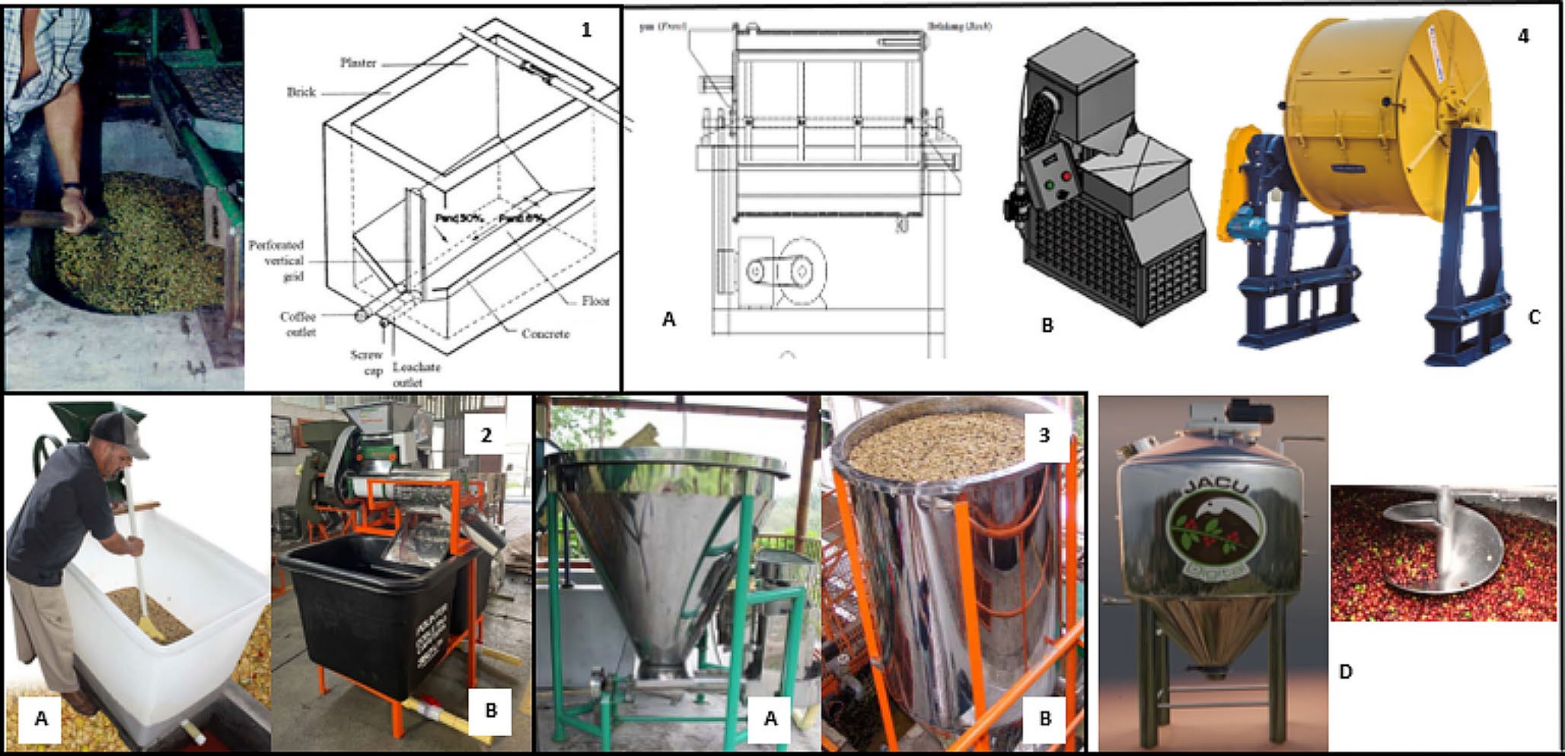
(**1**) Vat-type tank for fermentation of pulped coffee. (**2**) High-density polyethylene vat-type tanks for coffee fermentation. (**A**) Cenicafé (**B**) Rotoplast^®^. (**3**) Fermentation tank in Ecomill^®^ Technology. (**A**) 1,000 to 1,500 kg load capacity. (**B**) 2,000 to 6,000 kg load capacity. (**4**) Closed systems for coffee fermentation. (**A**) Widyotomo, S., & Yusi, Y. 2013, (**B**) Correa et al. 2014, (**C**) Palinialves, (**D**) CampoTech – Jacu Digital

Widyotomo, S., and Yusi, Y. (2013) evaluated the fermentation of cherry coffee in a horizontal type fermenter with electrical resistance and agitation (Fig. 4-4-A). Working at 50% capacity (20 kg/batch), temperatures between 20 and 40 °C and times between 6 and 18 h, the authors defined optimal operating conditions at 25 °C and 12 h of processing. In an attempt to remove the mucilage using low temperatures to reduce the consumption of washing water, Bressani et al. (2020) evaluated a cold fermenter prototype. Temperatures close to 2 °C managed to denature the structure of the mucilage and then it was removed mechanically (Fig. 4-4-B). In Brazil, some private companies have developed commercial prototypes for fermentation control. The Palinialves company developed a rotating cylinder (Fig. 4-4-C) with a galvanized sheet, steel or stainless steel structure with internal blades and rpm control for a homogeneous mix. The system is completely sealed and has a relief valve for pressure control and temperature sensors. Its maximum capacity is 10,000 L. The Campotech company with the support of Embrapa and the Instituto Federal do Sul de Minas, developed a device for the controlled fermentation of 1,250 L of cherry or pulped coffee. The device, in the form of a vertical cylinder and conical base, has a helical agitation and temperature control systems for heating or cooling the coffee mass (Fig. 4-4-D).

## Final remarks

Colombia, with over a century of coffee production experience, has limited knowledge in developing innovative fermentation prototypes. The fermentation process for washed coffees was once considered unimportant, focusing only on removing mucilage to reduce drying time. This neglect of safety and quality has led to issues with materials like concrete, majolica, wood, and cement. Technological advances in the last decade have led to the use of safe materials like high-density polyethylene and stainless steel in fermenters. Prototype fermenters or bioreactors with variable control systems and mechanical agitation have been developed in countries like Peru, Brazil, Chile, Spain, Indonesia, and Colombia. However, these high-cost technologies remain inaccessible to most producers. The industry has developed solutions such as helical-type central agitators and rotating drums, both with high energy consumption. A strategy is being evaluated for mixing through the recirculation of leachate, which requires less energy than the entire coffee mass. However, the impact of this methodology on the process quality has not been scientifically evaluated (Widyotomo and Yusianto 2013).

## Conclusions

A bibliometric analysis of the literature on coffee fermentation indicates a growing interest and progress in research and development of technologies to improve sensory quality, safety, efficiency, and sustainability. Improvements in fermentation tanks have been identified in terms of materials, designs, and the incorporation of accessories such as digital sensors. Innovations in fermentation methodologies and a more scientific approach by researchers in this field have also been observed.

Moreover, the analysis indicates that issues related to coffee bean fermentation are undergoing constant evolution, with Brazil emerging as a leading contributor in this field. Despite a decline in the number of published papers over the past three years, research is focused on the design of controlled fermentations and the evaluation of the influence of microorganisms and process conditions on the sensory quality and composition of coffee. Nevertheless, it is observed that prototypes designed to regulate process variables, such as agitation and temperature, are costly and may be inaccessible to small-scale producers.

Collectively, these findings indicate that the integration of innovative technologies, enhanced methodologies, and a rigorous scientific approach is transforming the coffee industry towards enhanced efficiency, safety, and sustainability, with the potential to benefit both producers and consumers globally.

## Data Availability

Not applicable.
